# Physical activity in the first postoperative week in 132 knee arthroplasty patients randomized to 3 different analgesic regimens

**DOI:** 10.1097/MD.0000000000033471

**Published:** 2023-04-21

**Authors:** Vigdis Schnell Husby, Torbjørn Rian, Jomar Klaksvik, Tina Strømdal Wik, Siri Bjørgen Winther

**Affiliations:** a Department of Orthopaedic Surgery, Trondheim University Hospital, Trondheim, Norway; b Department of Health Sciences Aalesund, Faculty of Medicine and Health Science, Norwegian University of Science and Technology, Aalesund, Norway; c Clinic of Anesthesia and Intensive Care, Trondheim University Hospital, Trondheim, Norway; d Department of Neuromedicine and Movement Science, Faculty of Medicine and Health Science, NTNU, Norwegian University of Science and Technology, Trondheim, Norway; e Orthopedic Research Center, Orthopaedic Department, Trondheim University Hospital, Trondheim, Norway.

**Keywords:** accelerometer, arthroplasty, knee, mobilization, physical activity

## Abstract

The aim of this study was to investigate whether objectively recorded physical activity in the first week after surgery in total knee arthroplasty patients differed between patients allocated to 3 different analgesic regimens.

**Methods:**

A total of 132 total knee arthroplasty patients wore activity monitors 24 hours a day from day 1 after surgery for 6 consecutive days. The time mobilized (stepping/standing) and the number of steps were recorded. This study was a sub-study of a randomized controlled study comparing tapentadol extended-release (ER), oxycodone controlled-release (CR), or a non-opioid placebo analgesic regimen.

**Results:**

The placebo group spent significantly more time mobilized than the tapentadol ER and the oxycodone CR groups (*P* = .016 and .042, respectively), but no statistically significant differences were found between the groups in the number of steps taken. The activity levels of patients in all groups increased in the first week after surgery.

**Conclusion:**

Patients in the non-opioid placebo group spent more time mobilized the first week after surgery than those in the tapentadol ER and the oxycodone CR groups, while the number of steps was not different between the groups.

**What is known?**

• Patients undergoing total knee arthroplasty (TKA) experience moderate-to-severe pain after surgery, which could negatively influence early mobilization and discharge to home. Early mobilization after surgery is important to avoid complications.

**What is new?**

• The study compared objectively measured early physical activity in TKA patients allocated to 3 different analgesic regimens. The present study contributes to increased knowledge about what could be considered “normal” activity levels early after TKA surgery. As hospital length of stay has decreased, it is important to get knowledge of how the patients cope at home concerning physical activity.

## 1. Introduction

Early mobilization after surgery is strongly recommended to avoid postoperative complications,^[1]^ regain independence, and quickly return to everyday activity. Early mobilization is defined as getting out of bed, standing, and/or walking as soon as possible after surgery.^[2]^ Most studies have objectively assessed physical activity in total knee arthroplasty (TKA) patients from 6 weeks to 4 years after surgery.^[3–7]^ The studies reported small improvements in physical activity after surgery compared to preoperative levels of activity, and less than the recommended levels of 150 to 300 minutes a week of moderate-intensity physical activity for the healthy adult population.^[8]^ However, the level of physical activity in the early postoperative phase has not been studied extensively. The relationship between postoperative pain and activity level remains unclear; a study recording physical activity in 40 TKA patients at 3 weeks after surgery found considerable variations between the patients and that pain was only weakly associated with physical activity.^[9]^ Another small study of TKA patients recorded physical activity 1 to 2 weeks postoperatively and found reduced physical activity, which was not influenced by pain.^[10]^

Severe postoperative pain occurs in up to 60% of patients receiving TKA.^[11]^ Long-lasting pain (>3 months) affects 7 to 20% of TKA patients^[12]^ and intense postoperative pain is associated with a higher risk of long-lasting pain.^[13]^ In addition to patient discomfort, pain also leads to increased hospital length of stay and reduced early postoperative ambulation, which in turn may cause thromboembolism, reduced knee range of motion, prolonged rehabilitation,^[11]^ and functional problems.^[14]^

To counteract moderate and severe postoperative pain after TKA, nerve blockade, infiltration analgesia,^[15]^ and various combinations of analgesics and anti-inflammatory drugs have been introduced. Furthermore, opioid-sparing multimodal analgesic regimens have been developed to avoid the side effects of opioids that could hinder early physical activity.

The aim of the present study was to investigate whether objectively measured physical activity differed between patients allocated to a tapentadol extended-release (ER) group, an oxycodone controlled-release (CR) group, and a non-opioid placebo analgesic regimen group in the first week after TKA.

## 2. Methods

### 2.1. Patients

One hundred forty-nine patients scheduled for primary TKA at St. Olavs hospital, Trondheim University Hospital, Norway, were included in the study. The inclusion took place between November 26, 2015, and November 7, 2018. Patients were recruited by an anesthesiologist at the outpatient preoperative clinic. The inclusion criterion was an age of 18 to 80 years. The exclusion criteria were cognitive impairment, not reading/speaking Norwegian, no cellphone or wireless Wi-Fi connection at home, use of drugs (e.g., antidepressive drugs or angiotensin II receptor blockers), or medical conditions that, according to the Norwegian Pharmaceutical Compendium, conflicted with one or more of the study drugs or any of the multimodal basal pain medications given in the study. Patients with regular opioid use or previous opioid abuse were excluded. Pregnant or breastfeeding women and patients scheduled for general anesthesia were not included in the study.

### 2.2. Study design

This was a sub-study of a prospective, randomized, double-blind placebo-controlled study.^[16]^ The patients underwent TKA (NexGen CR; Zimmer Biomet, Warsaw, Indiana) (132 of 134 patients) or patellofemoral arthroplasty (Gender; Zimmer Biomet) (2 of 134 patients). Surgery was performed with or without the use of a tourniquet at the surgeon’s preference (Table 1). The tourniquet pressure was set at 100 mm Hg above the systolic blood pressure.

**Table 1:** Demographics and preoperative characteristics of the patients.

Group	Oxycodone CR (n = 46)		Tapentadol ER (n = 44)		Placebo (n = 42)	
Sex, F/M Tourniquet use TKA/PFA	28/18 23 46/0		22/22 26 43/1		20/22 30 41/1	
	Mean (SD)		Mean (SD)		Mean (SD)	
Tourniquet time (min)	54.6	(35.1)	48.7	(35.4)	62.0	(30.5)
Surgery time (min)	84.7	(20.8)	87.7	(22.2)	91.2	(22.0)
Age	61	(9)	60	(9)	63	(11)
BMI	29.5	(4.1)	28.8	(4.8)	28.0	(4.0)
ASA-score	2	(1)	2	(1)	2	(1)
NRS mobilization (0–10)	5.3	(1.9)	5.0	(2.3)	5.7	(2.4)
NRS rest (0–10)	2.5	(2.2)	2.3	(1.9)	2.3	(2.5)
Charlson Comorbidity index (CCI), age-adjusted	2.2	(1.3)	2.0	(2.2)	2.2	(1.3)

Sex, tourniquet use, and TKA/PFA are presented as counts.

ASA = American Society of Anesthesiologist score, BMI = Body Mass Index, CR = controlled-release, ER = extended-release, F = female, M = male, NRS = numeric rating scale, PFA = patellofemoral arthroplasty, TKA = total knee arthroplasty.

### 2.3. Randomization and interventions

Patients were randomly assigned to 1 of 3 study groups, receiving either oxycodone CR 10 mg × 2, tapentadol ER 50 mg × 2, or placebo as their study drug in addition to multimodal basal pain medications. Details of the randomization method and intervention have been described previously.^[16]^ In this double-blind study only the hospital pharmacy, the monitor unit, and the manufacturer of the study drugs knew which study drugs the patients received, and each patient got a unique study code that could be unblinded in case of emergency.^[16]^

Naproxen, acetaminophen, and the study drug were given as scheduled drugs for 7 days. Thereafter, the patients were advised to use naproxen 500 mg and esomeprazole 20 mg twice daily, acetaminophen 1000 mg up to 4 times a day, and oxycodone immediate-release (IR) 5 mg if needed. The study was finalized with a telephone interview 14 days postoperatively. All patients followed a standardized fast-track patient course as previously described in the literature.^[17]^ On the day of surgery, all patients received acetaminophen tablets (2/1.5 g above/below 70 kg), dexamethasone tablets (20/16 mg above/below 70 kg), naproxen (500 mg) + esomeprazole (20 mg, 1 tablet) and the study drug. All patients were anesthetized using spinal anesthesia (bupivacaine 5 mg/mL, 2.5 mL) and sedated with propofol during surgery. All patients received local anesthetic infiltration with ropivacaine 2 mg/mL, 100 mL at the end of surgery.

When the patients arrived at the recovery unit, an activity monitor (activPAL™, PAL Technologies Ltd., Glasgow, UK) was placed on the patient’s non-operated thigh before mobilization. The patients were mobilized using a pulpit walker as soon as the motor function/control of the lower extremities was adequate. All patients received daily individual physical therapy during their hospital stay that included instructions for stretching, getting in/out of bed, walking with crutches on plain surfaces as well as ascending/descending stairs. A daily group session led by a physiotherapist was optional.

From the day of surgery, the patients received study medication twice daily. The multimodal basic pain medication consisted of naproxen 500 mg and esomeprazole 20 mg 1 tablet twice daily and acetaminophen 1000 mg 4 times daily for 7 days. Study drugs were given at the same time of the day during hospitalization, which corresponded to the nurses’ medication rounds. After discharge, pain medication times were at the patient’s own preference. The instructions were identical for all patients with morning, noon, afternoon, and evening as “medication times.” No instructions for pain medication by clock times were given. For rescue analgesia, patients had the opportunity to take 5 mg oral oxycodone IR on demand. The use of all scheduled analgesics, study medication, and daily doses of rescue oxycodone were recorded. The use of all scheduled analgesics, study medication, the daily dose of rescue oxycodone as well as registration of pain level and side effects were recorded by the patients using a tablet/iPad with an application developed for the study.^[18]^

#### 2.3.1. Daily physical activity.

Daily physical activity was assessed using a small, body-worn, single-axis, accelerometer-based activity monitor. The start and duration of monitoring were preset, and the monitor was attached to the patients’ non-operated thigh using double-sided hydrogel adhesive pads and covered with waterproof tape. The monitor was worn continuously for 24 hours a day for 7 days. Activity monitoring data from midnight after surgery and 6 consecutive days were used for analysis.

Physical activity was reported as time mobilized, meaning the time the patient spent standing or stepping, and the daily number of steps for the first 6 postoperative days.

The activPAL^TM^ monitor is a valid and reliable measure of walking time, posture, and motion during everyday physical activity.^[19,20]^

#### 2.3.2. Pain.

Pain at rest and during mobilization was reported daily using the numeric rating scale (NRS), where 0 indicated no pain and 10 indicated the worst pain imaginable.

### 2.4. Data analysis

The sample size was based on the primary outcome NRS from the main study.^[16]^ A general linear mixed model was used to analyze repeated measures of time mobilization and step count during the first 6 postoperative days. The outcomes were modeled with groups as fixed factors and with a random subject intercept. Outcomes were adjusted for body mass index, age, and tourniquet (yes/no). The normality of the residuals was verified using histograms. The group differences presented in the results section represent model estimates. The statistical analysis was per per-protocol. Statistical analyses were performed using the SPSS version 27 software (IBM, Armonk, NY). Statistical significance was set at *P* < .05.

### 2.5. Ethical considerations

The study was approved by the regional ethics committee (2015/209 REK) and the Norwegian Medicines Agency (SLV) (15/01581-13) and was registered at ClinicalTrials.gov (NCT02604446). All authors from the main study approved the ethical protocol for the sub-study. The study was conducted in accordance with the Declaration of Helsinki and Good Clinical Practice guidelines. Written informed consent was obtained from all subjects. This study was monitored by The Norwegian University of Science and Technology, Unit for Applied Clinical Research. This study conforms to all CONSORT guidelines and reports the required information accordingly.

## 3. Results

**Figure 1.**
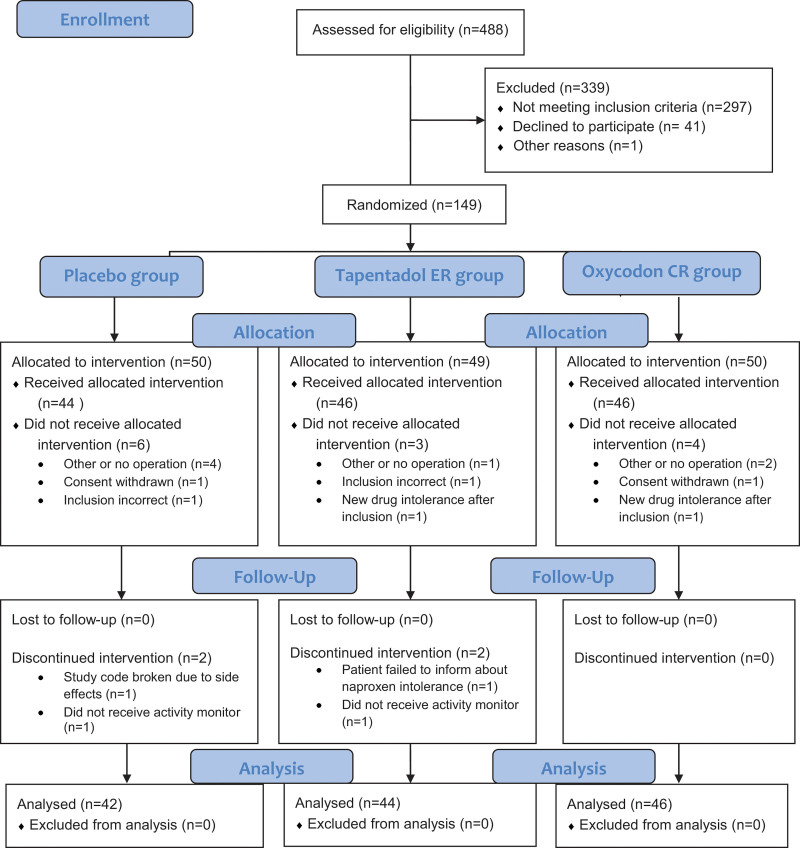
CONSORT 2010 flow diagram showing the patient flow through the study.

One hundred thirty-four patients received the allocated intervention, of which 132 patients had valid activity-monitoring data for the entire period. Patient flow through the study is shown in Figure 1. The demographic and preoperative characteristics of the patients are shown in Table 1.

Daily time mobilized, meaning the time spent stepping and standing, was 0.6 hours longer in the placebo group compared to the tapentadol ER group (*P* = .016), and 0.5 hours longer than the oxycodone CR group (*P* = .042) (Table 2). The time standing contributed the most to the differences in time mobilized.

**Table 2:** Mean time mobilized and sitting/lying (hours) the first 6 days after TKA surgery.

Group	Oxycodone CR (n = 46)		Tapentadol ER (n = 44)		Placebo (n = 42)	
	Mean	95% CI	Mean	95% CI	Mean	95% CI
Time Sitting/lying (h)	21.26	(21.10–21.42)	21.25	(21.10–21.40)	20.66	(20.45–20.86)
Time Mobilized (h)	2.74	(2.58–2.90)	2.75	(2.60–2.90)	3.34	(3.14–3.55)

CI = confidence interval, CR = controlled-release, ER = extended-release, TKA = total knee arthroplasty.

**Figure 2.**
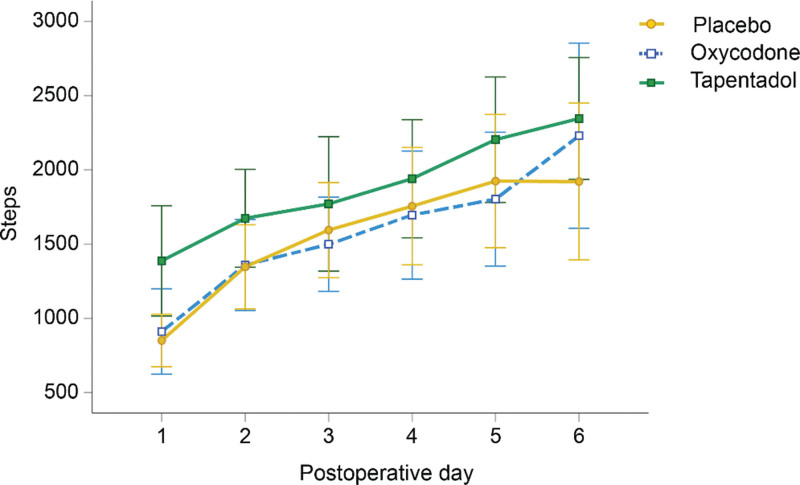
Step count days 1 to 6 postoperatively for the tapentadol extended-release (ER) group, the oxycodone controlled-release (CR) group, and the non-opioid placebo group.

The tapentadol ER group had a higher daily step count than the placebo and oxycodone CR groups (Fig. 2), but the group differences were not statistically significant (*P* = .331). The number of steps increased significantly in all groups from days 1 to 6, with an average daily increase of 206 steps (*P* < .001).

**Figure 3.**
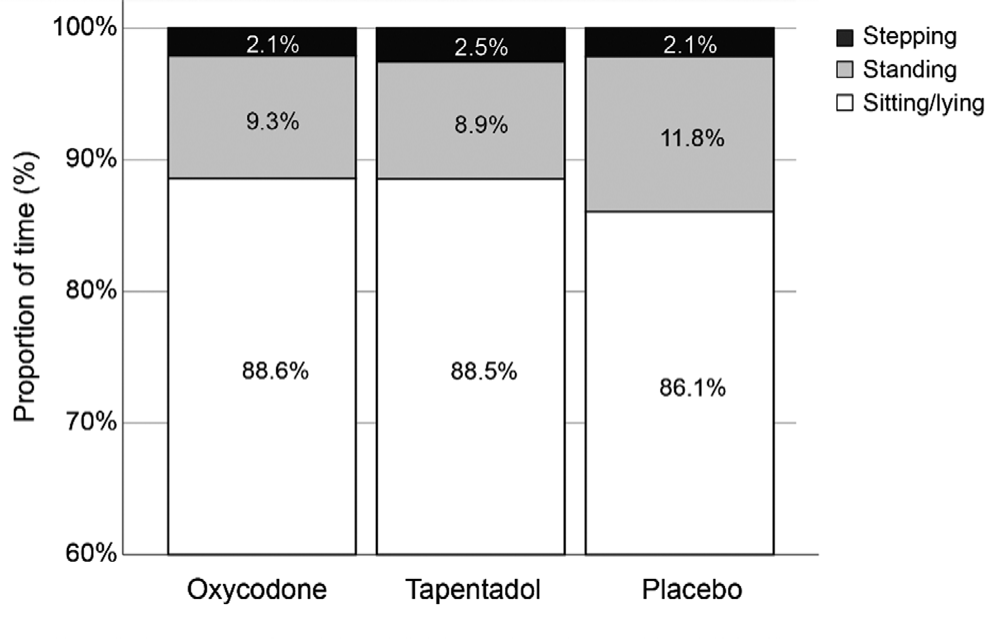
Proportion of time in walking, standing, and lying position during the first 6 postoperative days for the tapentadol extended-release (ER) group, the oxycodone controlled-release (CR) group, and the non-opioid placebo group.

For all patients in the study together, the mean time mobilized per day was 2.93 hours which increased by 0.13 hours per day from day 1 to day 6 (*P* ≤ .001). The patients were sitting or lying on average 88% of the time in the first 6 days after surgery (Fig. 3).

**Figure 4.**
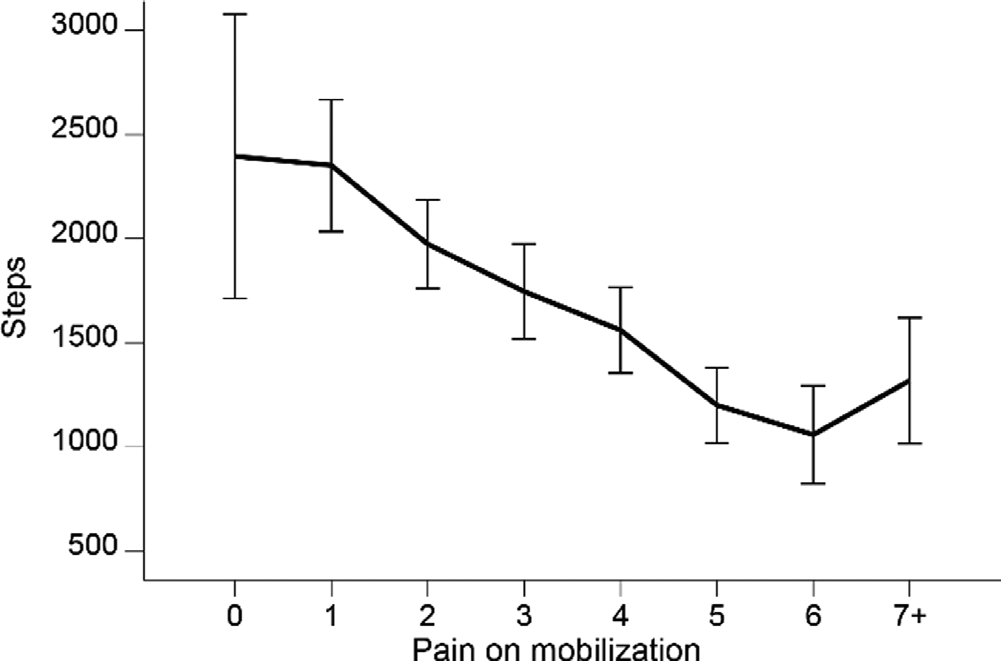
Pooled data showing number of steps for all groups from days 1 to 6 postoperatively and the association with pain mobilized on the NRS score. Pain on mobilization measured by NRS. NRS = numeric rating scale.

Pooling the data for all patients in the study for all 6 days, we found that the number of steps decreased with increasing pain until the pain was reported as severe (NRS 7 or more), after which the number of steps increased (Fig. 4).

Regarding drug intolerance, 1 patient in the placebo group was readmitted to the hospital 1 day after discharge due to hallucinations, probably because of too high an intake of oxycodone IR (<100 mg a day). This patient was excluded from the study.^[16]^

## 4. Discussion

We found that the placebo group spent significantly more time mobilized (standing and stepping) than the oxycodone CR and tapentadol ER groups, where time standing contributed the most to the increased time mobilized in the placebo group. Time mobilization and the number of steps per day increased in all groups in the first week after surgery. The step count was higher in the tapentadol ER group than in the other groups, but the difference did not reach statistical significance.

As reported in the main study, no statistical difference in pain during activity or use of rescue analgesics between groups receiving different analgesic regimens was found, although the placebo group showed the highest pain scores for both mobilization and rest.^[16]^ An explanation for the placebo group spending more time mobilized may be that various behavioral strategies, including standing, are activated to alleviate pain. Lunn et al^[21]^ studied the analgesic effects of exercise on the first day after TKA and found that walking reduced pain during the subsequent rest. The observed reduction in pain is suggested by the authors to be a result of the modulation of nociceptors during ambulation, which is probably also valid during standing, and thus may explain the increased time mobilized in the placebo group. Notably, the study by Lunn et al was a small pilot study without a control group. However, the findings are interesting, as non-pharmacological treatment could be a supplement to the multimodal analgesic treatment offered to TKA patients. Another possible explanation for the findings might be that the patients in the placebo group were more alert due to not experiencing the side effects of opioids. There were however no statistically significant differences between the groups regarding dizziness, sedation, and nausea (just a trend) as reported in the main study.^[16]^ Considering the opioid epidemic, the non-opioid analgesic regimen is of particular interest as 10.1 million people in the US misused prescription opioids in 2018.^[22]^ The present study is important as it reports on a non-opioid approach to alleviating postoperative pain in TKA patients. Further, keeping the focus on early mobilization is essential as immobilization is a well-known factor for postoperative complications, including thromboembolism.^[23]^

In the present study, the daily step count increased significantly in the first week in all groups. As demonstrated in Figure 2, the step count was higher in the tapentadol ER group than in the other groups, but the differences were not statistically different. In the main study, the mean number of daily steps for the 3 groups was 1887 in the tapentadol ER, 1596 in the placebo, and 1580 in the oxycodone CR, respectively.^[16]^ For comparison, Frimpong et al^[7]^ reported the daily mean number of steps to be 2093 at 6 weeks postoperatively after TKA. The number of steps reported in the study by Twiggs et al^[24]^ is below those reported in this study, as the TKA patients in their study took 1170 steps per day on days 2 to 4 after surgery. The patients in the study by Twiggs et al were discharged approximately 6 days after surgery, compared to 2 to 3 days in our previously presented results.^[16]^ Returning home usually increases activity and might explain these differences. The higher number of steps taken by the patients in the present study compared to those of Twiggs et al may be influenced by the fact that the patients in all groups in the present study followed a standardized fast-track regimen including a preoperative education program that encourages early physical activity. Twiggs et al did not report which analgesics were prescribed to the patients in their study or whether the patients were part of a standardized patient course.^[24]^ To the best of our knowledge, the number of steps taken by TKA patients in the first postoperative week has not been reported in other studies.

In the current study, we found that the number of steps decreased with increasing pain intensity when mobilized, but only to a certain point. The relationship between pain intensity and activity level remains unclear. Chan et al reported in their study of 171 TKA patients that pain negatively influenced walking 1 to 2 weeks after surgery.^[25]^ In contrast, Krenk et al reported no association between pain and activity.^[10]^ This result may be explained by the fact that this finding is based on pooled data for TKA patients and patients undergoing total hip arthroplasty and that recovery might differ between TKA and total hip arthroplasty patients.

Physical activity increased in all groups early after surgery in the present study. These results are consistent with those of Luna et al^[9]^ who found a daily increase of 0.7% in physical activity from days 1 to 9 postoperatively. Despite increased activity, the patients in the 3 groups in this study mobilized only 12% (2.93 hours) of the day. In comparison, the time mobilized per day 6 weeks after TKA surgery was 22% in the study by Frimpong et al.^[7]^ Fatigue after surgery is a well-known experience for patients. Surgical stress response, which induces hormonal and metabolic changes, is one of several factors that cause postoperative fatigue.^[10,26]^ Furthermore, the fear of increased pain when moving around may also prevent patients from being more active, as movement-evoked pain is higher than pain at rest.^[27]^ However, a study of physical activity in >2000 Canadian adults showed that the total time mobilized per day was approximately 4 hours.^[28]^ Hence, the time mobilized in TKA patients in this study did not seem to be low when considering the early period of rehabilitation.

The present study reports detailed information about physical activity early after surgery in TKA patients, which is beneficial knowledge to clinicians. Further, the results from the study imply that the regular use of depot opioids does not lead to superior physical activity in the first week after TKA surgery. This is also valuable information for patients that for various reasons do not tolerate opioids.

This study has some limitations. We had no preoperative physical activity data from the patients, and it would have been of interest to compare our findings with preoperative physical activity levels. Because only patients aged between 18 and 80 years were included in the study, the results may not be applicable to patients aged >80 years. Statistical analysis is per per-protocol in the present study. Considering the very low number of dropouts, we assume that the per-protocol method has not led to significant bias. The strength of the present study was the high number of patients with complete activity data for the entire recording period. Another strength is that the patients wore the activity monitors from midnight on the first day after surgery and for 6 consecutive days. All patients in the study followed a standardized fast-track patient course that minimized other treatment differences, in addition to the 3 analgesic regimens. The patients were randomized into groups to prevent selection bias. Objectively measured data on early physical activity in TKA patients are scarce, and the present study reporting data from 132 patients contributes to increased knowledge about what could be considered “normal” activity levels early after TKA surgery in patients aged <80 years. Furthermore, as hospital length of stay has decreased, it is important to understand how patients cope with physical activity at home.

In conclusion, the placebo group spent more time mobilized (standing and stepping) than the ER and CR groups, but there were no differences in the number of steps between the groups. All patients showed increased activity from days 1 to 6 after surgery. This study contributes to increased knowledge about the expected activity level in the first week after TKA surgery.

## Author contributions

**Conceptualization:** Torbjørn Rian, Tina Strømdal Wik.

**Data curation:** Vigdis Schnell Husby, Torbjørn Rian, Siri Bjørgen Winther.

**Formal analysis:** Jomar Klaksvik.

**Project administration:** Torbjørn Rian, Tina Strømdal Wik.

**Writing – original draft:** Vigdis Schnell Husby.

**Writing – review & editing:** Torbjørn Rian, Jomar Klaksvik, Tina Strømdal Wik, Siri Bjørgen Winther.
